# Neurologic Disorders in Immunocompetent Patients with Autochthonous Acute Hepatitis E

**DOI:** 10.3201/eid2111.141789

**Published:** 2015-11

**Authors:** H. Blasco Perrin, P. Cintas, F. Abravanel, R. Gérolami, L. d'Alteroche, J.-N. Raynal, L. Alric, E. Dupuis, L. Prudhomme, E. Vaucher, P. Couzigou, J.-M. Liversain, C. Bureau, J.-P. Vinel, N. Kamar, J. Izopet, J.-M. Peron

**Affiliations:** Hôpital Purpan–CHU Toulouse, Toulouse, France (H. Blasco Perrin, P. Cintas, F. Abravanel, L. Alric, C. Bureau, J.-P. Vinel, J. Izopet, J.M. Peron);; Hôpital de La Conception–CHU Marseille, Marseille, France (R. Gérolami);; Hôpital Trousseau–CHU Tours, Tours, France (L. d'Alteroche);; Clinique de l’Union, l’Union, France (J.-N. Raynal);; Clinique Pasteur, Toulouse (E. Dupuis);; CHIC Castres-Mazamet, Castres-Mazamet, France (L. Prudhomme);; CH Narbonne, Narbonne, France (E. Vaucher);; Hôpital du Haut-Lévêque–CHU Bordeaux, Bordeaux, France (P. Couzigou);; CH Mont de Marsan, Mont de Marsan, France (J.-M. Liversain);; Hôpital Ranguei–CHU Toulouse, Toulouse (L. Buscail, N. Kamar)

**Keywords:** hepatitis E, hepatitis E virus, neurological manifestations, HEV, neurologic disorders, meningoradiculitis, immunocompetent, mononeuritis multiplex, Guillain-Barré syndrome, Parsonage–Turner syndrome, demyelinating neuropathy, viruses, *Suggested citation for this article*: Blasco Perrin H, Cintas P, Abravanel F, Gérolami R, d’Alteroche L, Raynal JN, et al. Neurologic disorders in nonimmunocompromised patients with autochthonous acute hepatitis E. Emerg Infect Dis. 2015 Nov [*date cited*]. http://dx.doi.org/10.3201/eid2111.141789

## Abstract

Neurologic disorders, mainly Guillain-Barré syndrome and Parsonage–Turner syndrome (PTS), have been described in patients with hepatitis E virus (HEV) infection in industrialized and developing countries. We report a wider range of neurologic disorders in nonimmunocompromised patients with acute HEV infection. Data from 15 French immunocompetent patients with acute HEV infection and neurologic disorders were retrospectively recorded from January 2006 through June 2013. The disorders could be divided into 4 main entities: mononeuritis multiplex, PTS, meningoradiculitis, and acute demyelinating neuropathy. HEV infection was treated with ribavirin in 3 patients (for PTS or mononeuritis multiplex). One patient was treated with corticosteroids (for mononeuropathy multiplex), and 5 others received intravenous immunoglobulin (for PTS, meningoradiculitis, Guillain-Barré syndrome, or Miller Fisher syndrome). We conclude that pleiotropic neurologic disorders are seen in HEV-infected immunocompetent patients. Patients with acute neurologic manifestations and aminotransferase abnormalities should be screened for HEV infection.

## Abstract 2

Patients with acute neurologic manifestations and aminotransferase abnormalities should be screened for hepatitis E virus infection.

Hepatitis E virus (HEV) infection is an emerging autochthonous disease in industrialized countries (*1*). Locally acquired (autochthonous) HEV 3 or HEV 4 infections are believed to be a porcine zoonosis. The virus typically affects middle-aged or elderly men and can cause severe hepatitis, particularly in patients with an underlying liver disease (*2*)*.* Chronic HEV infection occurs in recipients of a solid-organ transplant, in patients with hematologic malignancies, and in patients with HIV infection (*3**,**4*). Neurologic symptoms have been reported in up to 5% of patients with an HEV infection, indicating that HEV could have a specific neurotropism (*5*); most patients in this preliminary study were immunosuppressed. In developing countries, cases describing neurologic involvement during acute HEV infection have also been reported (*6**,**7*): most concerned Guillain-Barré syndrome (GBS) and Parsonage-Turner syndrome (PTS). Peripheral neuropathy, small-fiber neuropathy, and myositis have also been described (*8**,**9*). Peripheral nervous system tropism is not exclusive, and rare cases of myelitis and encephalitis have also been reported (*10*). Recently, studies of 2 national cohorts (in the United Kingdom and the Netherlands) have found that GBS and PTS were associated with acute HEV infection in 5% and 10% of cases, respectively (*11**,**12*)*.* Here we describe an additional range of neurologic manifestations that were found during acute autochthonous HEV infection in immunocompetent patients.

## Patients and Methods

This retrospective multicenter study was conducted in France from January 2006 through June 2013. All members of the French Liver Association (Association Française pour l’Etude du Foie, 500 members) were sent a newsletter inviting them to report cases of HEV infection in which neurologic symptoms were experienced. Data were recorded from patients with neurologic disorders, regardless of the symptoms exhibited during the course of acute HEV infection. This retrospective sampling was likely to capture most patients in France who had neurologic symptoms during acute HEV infection. A second newsletter was sent 6 months later to maximize the completeness of reporting. HEV infection was diagnosed by detecting anti-HEV IgM, HEV RNA, or both in serum or fecal samples from patients with elevated aminotransferase levels. All cases occurred in France, and all patients gave their informed consent.

The EIAgen (Adaltis, Eurobio, Bologna, Italy) and Wantai (Wantai Biologic Pharmacy Enterprise Co., Beijing, China) kits were used to detect antibodies (*13*)*.* Both tests are highly specific and sensitive for detecting IgM against HEV. HEV RNA from serum, fecal samples, or cerebrospinal fluid (CSF) was detected by using a real-time PCR as described (*3**,**14*)*.* All other causes of acute hepatitis were excluded, and patients had no co-infections. All patients were screened for other causes of disease with immunoassays (antinuclear antibodies, antipolynuclear neutrophil cytoplasmic antibodies) and underwent viral serologic testing (hepatitis B virus, hepatitis C virus, HIV, Epstein-Barr virus, cytomegalovirus). *Campylobacter* was also screened for. The cases of patients 5 and 12 have been reported (*14*).

Phylogenetic analyses were performed by sequencing a 347-nt fragment within the open reading frame 2 region as described (*15*). Nucleotide identity was analyzed with BioEdit software version 7.0.9.0 (http://www.mbio.ncsu.edu/BioEdit/bioedit.html).

### Statistical Analyses

Data are presented as their medians (ranges) or their means (SDs). Student *t*-test was used to compare the quantitative variables. A p value <0.05 was considered significant.

## Results

### Patient Characteristics and Clinical Outcomes

Fifteen patients (7 women, 8 men) from 7 different hospitals were included in this study. The clinical characteristics of the patients are reported in Table 1. The median age was 55 years (range 25–77 years). The median follow-up period was 42 weeks (range 4–161 weeks). All patients were immunocompetent. All but 1 lived in southern France: 11 lived in the southwest, 3 in the southeast, and 1 in central France.

**Table 1:** Clinical characteristics of 15 patients with hepatitis E virus–linked neurologic disorders, France, January 2006–June 2013*

Patient no.	Neurologic pathology	Age, y/sex	Treatment	Other symptoms	Neurologic symptoms at last follow-up	Follow up times, wk
1	MM	59/M	None	Asthenia	No	10
2	MM	44/F	None	Fever, asthenia, arthromyalgia	Yes	4
3	MM	65/M	Corticosteroids	Asthenia	No	157
4	MM	25/F	None	None	No	33
5	MM	49/M	None	None	No	NA
6	MM	77/F	RBV	None	No	44
7	PTS	51/M	RBV + IVIg	Arthromyalgia, asthenia	No	100
8	PTS	55/F	None	Asthenia, arthromyalgia	Yes	126
9	PTS	56/M	None	Asthenia	Yes	42
10	PTS	56/M	RBV + IVIg	Asthenia	Yes	16
11	MR	74/M	IV Ig	Arthromyalgia, asthenia	No	77
12	MR	54/F	None	Fever, nausea	No	NA
13	MR	33/F	None	Fever, arthromyalgia	No	161
14	GBS	60/F	IV Ig	Asthenia, acute low back pain	Yes	5
15	MFS	54/M	IV Ig	Asthenia, anorexia	Yes	13

*MM, mononeuritis multiplex; PTS, Parsonnage-Turner syndrome; MR, meningoradiculitis; GBS, Guillain–Barré syndrome; MFS, Miller-Fisher syndrome; RBV, ribavirin; IVIg, intravenous immunoglobulin; NA, not available.

No patient had underlying chronic liver disease; 2 patients had jaundice, 10 had asthenia, 5 had arthromyalgia, and 3 had fever. Three patients had neurologic symptoms only. Their biological characteristics are reported in Table 2. All patients had elevated liver enzyme levels, and none experienced liver failure. Median enzyme levels at diagnosis were the following: alanine aminotransferase level at diagnosis was 495 IU/L (range 49–3,641 IU/L; reference range 10–41 IU/l), aspartate aminotransferase 124 IU/L (range 37–1,742 IU/L; reference range 15–41 IU/l), bilirubin 15 µmol/L (range 4–101 µmol/L; reference range 3–21 µmol/l), alkaline phosphatase 254 IU/L (range 88–704 IU/L; reference range 36–126 IU/l), γ-glutamyl transferase 185 IU/L (range 33–783 IU/L; reference range 7-64 IU/l). Median prothrombin time was 96% (range 76%–100%).

**Table 2:** Liver test results and virologic characteristics of 15 patients with hepatitis E virus–linked neurologic disorders, France, January 2006–June 2013*

Patient no.	Diagnosis	AST, IU/L	ALT, IU/L	Bilirubin, µmol/L	ALKP, IU/L	GGT, IU/L	PT, %	GT	Serology	Serum viral load, log-copies/mL	PCR stools	PCR CSF
1	MM	69	256	8.6	254	323	92	3f	IgG+/IgM+	4.92	NA	NA
2	MM	507	756	17	540	187	90	3f	IgG+/IgM+	5	Pos	NA
3	MM	NA	3,641	90	NA	NA	80	NA	IgG+/IgM+	Neg	Neg	NA
4	MM	54	120	10	NA	95	100	NA	IgG+/IgM+	Neg	Neg	NA
5	MM	50	118	14	90	95	95	NA	IgG+/IgM+	Pos	NA	Neg
6	MM	71	119	14	430	131	100	3f	IgG–/IgM+	5.57	Pos	NA
7	PTS	897	1,834	42	336	383	88	NA	IgG+/IgM+	2.12	NA	NA
8	PTS	1.330	1,900	15	194	182	95	NA	IgG+/IgM+	2.81	NA	NA
9	PTS	601	1,376	15	231	601	95	NA	IgG+/IgM+	NA	NA	NA
10	PTS	135	495	17	659	740	100	3f	IgG+/IgM+	3.89	Pos	NA
11	MR	1.742	822	101	704	528	76	3f	IgG+/IgM+	5.83	Pos	Pos
12	MR	221	566	11	124	184	100	NA	IgG+/IgM+	Pos	NA	Pos
13	MR	100	246	4.7	248	33	98	3f	IgG–/IgM+	2.9	Pos	Neg
14	AIDP	113	384	34.2	474	783	100	3f	IgG+/IgM+	Pos	Pos	Neg
15	AIDP	37	49	7	88	48	94	NA	IgG+/IgM+	Neg	Neg	Neg

*AST, aspartate aminotransferase; ALT, alanine aminotransferase; ALKP, alkaline phosphatase; GGT, γ-glutamyl transpeptidase; PT, prothrombin time; GT, genotype; CSF, cerebral spinal fluid; MM: mononeuritis multiplex, NA, not available; Pos, positive; Neg, negative; PTS: Parsonage-Turner syndrome; MR: meningoradiculitis; AIDP, acute inflammatory demyelinating polyradiculoneuropathy.

All patients had IgM against HEV; 13 had IgG against HEV and 2 did not. Serum specimens of 14 patients underwent HEV PCR, and specimens from 11 patients were positive for HEV (Table 2). The median plasma HEV RNA concentration was 4.40 log-copies/mL (range 2.12–5.83).

We were able to sequence 7 virus strains from the patients: all belonged to genotype 3f, the main genotype identified in France. Phylogenetic analyses were conducted with these strains and HEV strains obtained by the French National Reference Center from patients who did not exhibit a neurologic disorder (Figure). The strains were scattered across the phylogenetic tree and were only 87.0%–93.5% identical.

Two patients had eaten game (wild boar and deer). All other patients had eaten pork. All patients had liver enzyme levels within the reference range at the last follow-up.

### Neurologic Symptoms

Fourteen patients had been hospitalized. Neurologic symptoms were classified into 4 categories: mononeuritis multiplex, PTS, meningoradiculitis, and acute inflammatory demyelinating polyradiculoneuropathy (Table 3).

**Table 3:** Average biological values of the 4 groups of patients with hepatitis E virus–linked neurological disorders*

Disorder	Age, y (range)	Sex ratio	AST, IU/L (± SD)	ALT, IU/L (± SD)	PT, % (± SD)	Bilirubin, μmol/L (± SD)	ALKP, IU/L (± SD)	GGT, IU/L (± SD)
MM	54 (25–77)	1	150 (± 200)†	835 (± 1,396)	92.8 (± 7.5)	25.6 (± 32)	328.5 (± 198)	166.2 (± 95)†
PTS	55 (51–56)	6	740.7 (± 503)	1,401.25 (± 647)	94.5 (± 4.9)	22.2 (± 13)	355 (± 211)	476.5 (± 245)
MR	54 (33–74)	2	687.7 (± 915)	544.67 (±288)	91.3 (± 13.3)	38.9 (± 54)	358.67 (± 305)	248.3 (± 253)
AIDP	57 (54–60)	1	75 (± 54)	216 (± 236)	97 (± 4)	20.6 (± 19)	281 (± 272)	415 (± 519)

*AST, aspartate aminotransferase; ALT, alanine aminotransferase; PT, prothrombin time; ALKP, alkaline phosphatase; GGT, γ-glutamyl transpeptidase; MM, mononeuritis multiplex; PTS, Parsonage-Turner syndrome; MR, meningoradiculitis; AIDP, acute inflammatory demyelinating polyradiculoneuropathy. †p<0.05 when compared with PTS.

#### Mononeuritis Multiplex

Six patients (3 men and 3 women, patients 1–6) had mononeuritis multiplex; their median age was 54 years (range 25–77 years). Mononeuritis multiplex was defined by asymmetric, asynchronous involvement of the noncontiguous nerve trunks. All patients had experienced neuropathic pain and paresthesia in >1 nerve segments with hyporeflexia or areflexia. For 3 patiens, an electromyogram showed asymmetric axonal neuropathy, and various patterns of nerve involvement were observed. Patients 1 and 6 had confluent but asymmetric (>50%) lower-limb neuropathy (musculocutaneous and external saphenous). Patients 3 and 4 had multiple radicular or proximal troncular neuropathies, and patient 5 had troncular median and internal brachial cutaneous-nerve involvement. Three patients had asthenia and 1 had jaundice; HEV RNA was detected by PCR in serum samples from 4 patients (patients 1, 2, 5, 6). 

One patient (patient 6) received specific antiviral treatment. He was given ribavirin, initially at a dose of 400 mg for 7 days (5.5 mg/kg/d) and was then given 600 mg/day (8.5 mg/kg/d) for 3 months. A serum specimen was negative for HEV after 10 days of treatment. Patient 3 was given corticosteroids for 10 weeks. Five patients had no sequelae at the last follow-up (median 33 weeks [range 4–157 weeks]). One patient (patient 2), at the last follow-up at 4 weeks, had ongoing paresthesia in a nerve segment in the lower limbs.

#### PTS

Four patients (3 men and 1 woman, patients 7–10) exhibited PTS, also known as neuralgic amyotrophy. Their median age was 55.5 years (range 51–56 years). These patients sought treatment at the hospital for asthenia and acute neuropathic pain in the shoulder. PTS was bilateral but asymmetric in 3 patients. Asymmetric paresis and amyotrophia appeared within a few days, with the concomitant decrease in pain. Tendon reflexes were reduced or eliminated in 2 patients (patients 9 and 10). For 3 patients, an electromyogram confirmed bilateral patchy denervation, predominantly of the upper trunk of the brachial plexus. For 2 patients (patients 7 and 10), analysis of CSF showed high levels of proteins, levels of cells in reference ranges, and glucose level within reference range. HEV PCR of a CSF specimen was not done.

HEV was diagnosed after IgM against HEV was detected in 1 patient (patient 9) and after HEV RNA was detected in the serum of 3 patients (patients 7, 8, 10). Patients 7 and 10 were given ribavirin (for, respectively, 3 weeks and 2 months) at a dose of 800 mg/day. This treatment was well tolerated. They also received intravenous immunoglobulin (IVIg) at a dose of 25 g/day (patient 7) or 35 g/day (patient 10) for 5 days. All patients but 1 had persistent weakness at the last follow-up (median 71 weeks [range 16–126 weeks]); none had received a control electromyogram. Assessment of patients through disability scales was not possible because this study was retrospective and involved many centers. Notably, the only asymptomatic patient at follow-up was the patient that received both ribavirin and IVIg.

#### Meningoradiculitis

Three patients (2 women and 1 man, patients 11–13) had meningoradiculitis. They were, respectively, 74, 54, and 33 years of age. These patients sought treatment for meningitis symptoms (headache, photophobia) and radiculitis with pain and paresthesia restricted to 1 or a few radicular topographies. CSF was clear and showed lymphocytic meningitis with >90% lymphocytes, a high level of protein (0.79–1.37 g/L, reference range 0.28–0.53 g/L), and glucose levels within reference ranges. Patient 11 had jaundice. PCR of all serum samples from all patients with meningoradiculitis showed HEV RNA. For 2 patients, PCR of CSF was positive for HEV (patients 11 and 12). One woman was breast feeding (patient 13); HEV was detected in her serum but not in breast milk. PCR detected HEV in fecal samples from 2 patients (patients 11 and 13). Patient 11 was treated with IVIg at 0.5 g/kg/day for 4 days. No patient was symptomatic at the last follow-up visit. The median time to follow-up was 119 weeks (range 77–161 weeks).

#### Acute Inflammatory Demyelinating Polyradiculoneuropathy

Two patients exhibited acute inflammatory demyelinating polyradiculoneuropathy: 1 had GBS, and 1 had Miller-Fisher syndrome. Patient 14, a 60-year-old woman, was hospitalized for GBS. Initially, she had asthenia and acute low back pain. She had a positive viral load for HEV IgG and IgM. A neurologic examination revealed generalized areflexia and weakness in the lower limbs. GBS was confirmed in nerve-conduction studies. She had no hepatic symptoms. CSF analysis showed a high level of protein (2 g/L, reference range 0.28–0.53 g/L) and no HEV RNA. She was treated with IVIg for 5 days at a dose of 400 mg/day, and the symptoms partially regressed. By 5 weeks later, weakness of the lower limbs had improved, but she still experienced persistent areflexia.

Patient 15, a 54-year-old man, was hospitalized for weight loss. A neurologic examination revealed quadridistal hypoesthesia with ataxia, areflexia, and diplopia due to paresia of the right VI nerve. CSF protein concentration was elevated (1.46 g/L, reference range 0.28–0.53 g/L), and albumino-cytologic dissociation was found. Nerve-conduction studies confirmed demyelinating neuropathy. These findings were conclusive for Miller-Fisher syndrome. He had no hepatic symptoms. Serum was positive for IgM against HEV. Serum and fecal samples were negative for HEV by PCR. He received IVIg for 5 days at a dosage of 2 g/kg and was hospitalized for 1 week. His neurologic symptoms improved but did not totally disappear until >13 weeks of follow-up (persistent ataxia).

## Discussion

This study shows the wide spectrum of neurologic injuries associated with patients with an acute HEV autochthonous infection. Because the study was retrospective, it was not possible to identify the prevalence of HEV infection in patients with neurologic symptoms or the prevalence of neurologic symptoms in patients with HEV infection. 

Neurologic symptoms could be divided into 4 entities. Notably, the first dominant finding was mononeuritis multiplex, observed in 6 of the 15 patients, with asymmetric, asynchronous, and painful segmental nerve involvement. This condition was also the main entity in the original report, which also included immunocompromised patients with chronic HEV-3 infection (*5*). In previous reports in immunocompetent patients, painful peripheral neuropathy has rarely been reported (*8*). However, mononeuritis multiplex is a common complication in immune and viral diseases. A vasculitis process can be hypothesized, but none of the patients in this study underwent a nerve biopsy. Additional investigations, including anatomopathologic exploration, would be needed to further explore the physiopathologic (antiviral and/or immunosuppressive) pathways.

The next most frequent manifestation was PTS, also called brachial neuritis or neuralgic amyotrophy, which was found in 4 patients. PTS is a rare pathologic condition, defined by sudden, acute, and unbearable pain across the top of the shoulder, followed by severe amyotrophy. As was exhibited by the patients described here, PTS can be unilateral or bilateral but is asymmetric. A recent study reported cases of acute HEV infection in a cohort of 47 patients from Cornwall (UK) and the Netherlands (*12*); 5 cases (10.6%) of acute HEV infection were identified, indicating that HEV may be a major cause of PTS in industrialized countries with a high prevalence of HEV. That study also suggested that HEV-associated brachial neuritis more commonly produces bilateral symptoms and signs than brachial neuritis produced by other causes. In our study, 3 of 4 patients had bilateral symptoms. Three of the 4 patients with PTS had neurologic sequelae at the last follow-up (71 weeks), which suggests that concomitant HEV infection may represent a poor prognostic factor. Van Eijk et al. (*12*) found that all 5 of their HEV-positive patients exhibited persistent weakness at 6 months.

There is no specific treatment identified for PTS, but it is known to be immune mediated. However, we cannot exclude direct infection of the brachial plexus. Three of the 4 patients in our study had serum samples positive for HEV by PCR, indicating active replication. Two patients were treated with ribavirin as an antiviral therapy. This antiviral therapy is recommended to treat chronic hepatitis E in organ-transplant recipients (*16**,**17*). Case reports on the treatment of acute HEV infection in immunocompetent patients have been published, with promising results (*18**–**21*). We found that the only patient who had no persisting symptoms at the last follow-up had been treated with a combination of ribavirin and IVIg. 

The third neurologic entity we found was meningoradiculitis in 3 patients. HEV was detected in the CSF of 1 case-patient, indicating neurotropism and the direct effect of HEV. This condition is probably the only one in which HEV may be directly responsible for the neurologic manifestations. No neurologic sequelae were seen in this group. A previous study found quasispecies compartmentalization in an immunocompromised patient with neurologic manifestations, and its temporal association suggests that neurologic symptoms could be linked to the emergence of neurotropic variants (*22*). The factors associated with HEV neurotropism need to be investigated further.

The last entity was acute inflammatory demyelinating polyradiculoneuropathy, which was seen in 2 patients. This condition can be classically triggered by many viruses, including hepatotropic viruses (*23*). An immune response that cross-reacts with axonemal or Schwann cell antigens is elicited and results in damage to the peripheral nerves. Both patients had persisting neurologic symptoms, commonly seen in this disease (*11*). Although several studies have demonstrated that HEV infection can induce GBS, this condition was found in only 2 of our patients. In a cohort of GBS case-patients from Bangladesh, seroprevalence of IgM against HEV was 11%, compared with 2% in a control group of patients with other neurologic disease (*7*). The frequency of HEV infection was recently determined in a cohort of 201 patients with GBS in the Netherlands and was found to have occurred in 5% (*11*).

The number of patients in this study is too small to draw definitive conclusions on the sex ratio for these conditions, but the ratio was highest in the PTS group. We also found that neurologic disorders occurred only in those with a HEV 3f genotype, although this is the most common genotype in France (*24*), so other HEV subtypes and genotypes may induce neurologic symptoms (*5*). Moreover, because only patients with elevated liver enzyme levels were included, neurologic complications were probably underestimated in this study, in particular, for GBS and PTS, for which conditions the neurologic symptoms are often delayed after the initial event is triggered.

Patients with PTS had significantly higher values in liver enzyme tests (aspartate aminotransferase, γ-glutamyl transpeptidase) than patients with mononeuritis multiplex. We have no definitive explanation, but this finding could be due to the earlier appearance of PTS symptoms during the course of HEV infection.

Three patients were treated with ribavirin with the aim of shortening the disease’s duration: the 2 PTS patients and 1 patient with mononeuritis multiplex. One needed blood transfusions and erythropoietin. Whether ribavirin shortened the evolution of neurologic symptoms, particularly for patients with PTS, needs to be studied further.

One limitation of our study is the retrospective sampling by emailing through the French Liver Association. Although this method provides the advantage of reaching most practitioners who diagnose HEV infection throughout France, it may have induced an ascertainment bias, that is, the true frequency of the neurologic symptoms during HEV infection is difficult to assess. In addition, patients with neurologic symptoms who were not referred to a hepatologist obviously were not accounted for.

In summary, HEV-3 infection can induce a wide range of neurologic symptoms, including mononeuritis multiplex, PTS, meningoradiculitis, and inflammatory demyelinating polyradiculoneuropathy. Sequelae were seen with mononeuropathy multiplex, PTS, and inflammatory demyelinating polyradiculoneuropathy. Only 2 patients had overt hepatitis with jaundice. Therefore, we recommend screening patients with elevated liver enzyme levels and neurologic symptoms for HEV infection, regardless of other symptoms. Treatment with ribavirin needs to be assessed further.
